# A Remote Health Monitoring System for the Elderly Based on Smart Home Gateway

**DOI:** 10.1155/2017/5843504

**Published:** 2017-10-24

**Authors:** Kai Guan, Minggang Shao, Shuicai Wu

**Affiliations:** College of Life Science & Bioengineering, Beijing University of Technology, Beijing, China

## Abstract

This paper proposed a remote health monitoring system for the elderly based on smart home gateway. The proposed system consists of three parts: the smart clothing, the smart home gateway, and the health care server. The smart clothing collects the elderly's electrocardiogram (ECG) and motion signals. The home gateway is used for data transmission. The health care server provides services of data storage and user information management; it is constructed on the Windows-Apache-MySQL-PHP (WAMP) platform and is tested on the Ali Cloud platform. To resolve the issues of data overload and network congestion of the home gateway, an ECG compression algorithm is applied. System demonstration shows that the ECG signals and motion signals of the elderly can be monitored. Evaluation of the compression algorithm shows that it has a high compression ratio and low distortion and consumes little time, which is suitable for home gateways. The proposed system has good scalability, and it is simple to operate. It has the potential to provide long-term and continuous home health monitoring services for the elderly.

## 1. Introduction

Recent years have witnessed China entering the aging society. The issue of population aging seems more and more serious. Physical conditions of the elderly, including cardiac function and the ability of maintaining gait balance, are declining. Health care and safety monitoring for the elderly is becoming an urgent issue to be solved.

The healthcare Internet of things (IoT) based on medical digital devices makes the home health monitoring for the elderly possible. By establishing an IoT-based home care monitoring system, the elderly can know about their health condition and get services provided by the health care center without walking out of home. It would also make the government and the society able to cushion the blow of the aging population.

In the home care monitoring system, the smart home gateway collects signals from the body sensor network (BSN) and transmits them to the health care server. The development of home gateway-based homecare monitoring systems has been through three stages. In the first stage, the telephone modem acts as the home gateway, and data was transmitted through the telephone line. Maiolo et al. [1] and Vitacca et al. [2] proposed monitoring systems by using modems for patients with chronic respiratory failure. This kind of health care systems can transmit a limited amount of data with a limited transmitting speed, which restricts the expansibility of the system. Meanwhile, when the data need to be transmitted, it needs to be manipulated by the patient, which seems not user-friendly. The popularization of the personal computer (PC) drives the health care monitoring system to its second stage. In the second stage, PCs were used as the home gateway, and data was transmitted through broadband. There is no doubt that PCs have enough operation ability to process data, while they consume large electric power. The third stage of health care monitoring system is characterized by embedded devices and smart devices [3–5]. Bansal et al. [6] and Jung et al. [7] proposed health care monitoring systems based on mobile gateways, like smart phones. Lin et al. [8] designed a set-top box-based homecare system. Spinsante and Gambi [9] proposed the TV-based mode of home care. Rahmani et al. [10] developed a smart home gateway and a corresponding monitoring system based on embedded technology; it can monitor multiphysiological signals of the elderly. Although each of these methods has their own advantages, there are also some drawbacks. The mobile gateway cannot guarantee long-term and continuous measurement. The set-top box-based system did not provide the measurement of ECG, which is the most important physiological signal for patients with cardiovascular diseases. Meanwhile, these solutions cannot guarantee 24 hours of monitoring for the elderly, and they are too sophisticated for the elderly to use.

Moreover, a huge amount of physiological data would generate during the process of health monitoring. It may cause problems of data overload and network congestion, which is a big burden for home gateway. The storing strategy of combining local storage and cloud storage proposed by Lin et al. [8] can reduce the storage pressure of the home gateway, but it consumes too much system resources when transmitting data. Previous studies [11–16] introduced the concept of data compression, which can reduce the amount of data effectively.

To implement long-term monitoring of the elderly, resolve issues of data overload and network congestion, and make it easy to operate for the elderly, a smart home gateway-based home care monitoring system was proposed in this paper. First, the system design is described. Then the principle and workflow of the ECG compression algorithm are presented. Lastly, the whole system and the compression algorithm are tested and evaluated.

## 2. Materials and Methods

### 2.1. System Design

**Figure 1:**
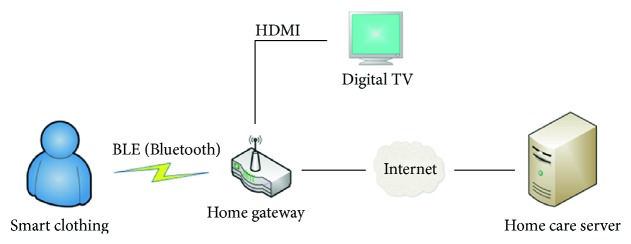
Architecture of the health monitoring system.

Figure 1 shows the architecture of the proposed system. The system comprises three parts, namely, the smart clothing for ECG and motion signals collection, the home gateway for data transmission, and the health care server for data storage and user information management.

Three-lead ECG signals and three-axis acceleration signals are obtained when the elders are wearing the smart clothing. The ECG signals are used to monitor the heart condition of the elderly, especially those with heart failure; the acceleration signals are used to monitor the body states of the elderly, such as walking and falling down. The smart clothing sends these signals to the home gateway via low-energy Bluetooth (BLE). And the home gateway gets data from the smart clothing through a Bluetooth connection. After local data processing, compression, and storage, the gateway transmits these data to the health care server via the Internet. The home gateway also provides service of video communication, which makes the connection between the elderly and doctors more convenient. The health care server is designed on the WAMP platform, and it is transplanted to the Ali Cloud® platform. It is used for long-term data storage and user information management.

#### 2.1.1. Wearable Smart Clothing

The ECG cables and the signals acquisition unit of smart clothing are designed separately. A cloth was designed with the ECG cables and the electrodes embedded in it. And the signal acquisition unit can be connected to the cloth through four metal buttons. The signal acquisition unit acquires three-lead ECG signals and three-axis acceleration signals and sends data to the home gateway via BLE. It is easy to operate and has low power consumption.

**Figure 2:**
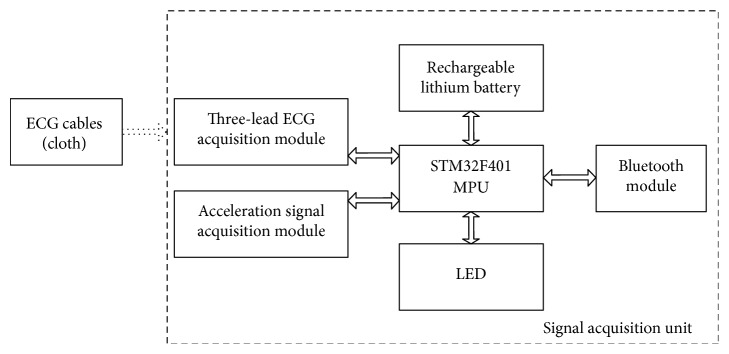
Hardware design of smart clothing.

**Figure 3:**
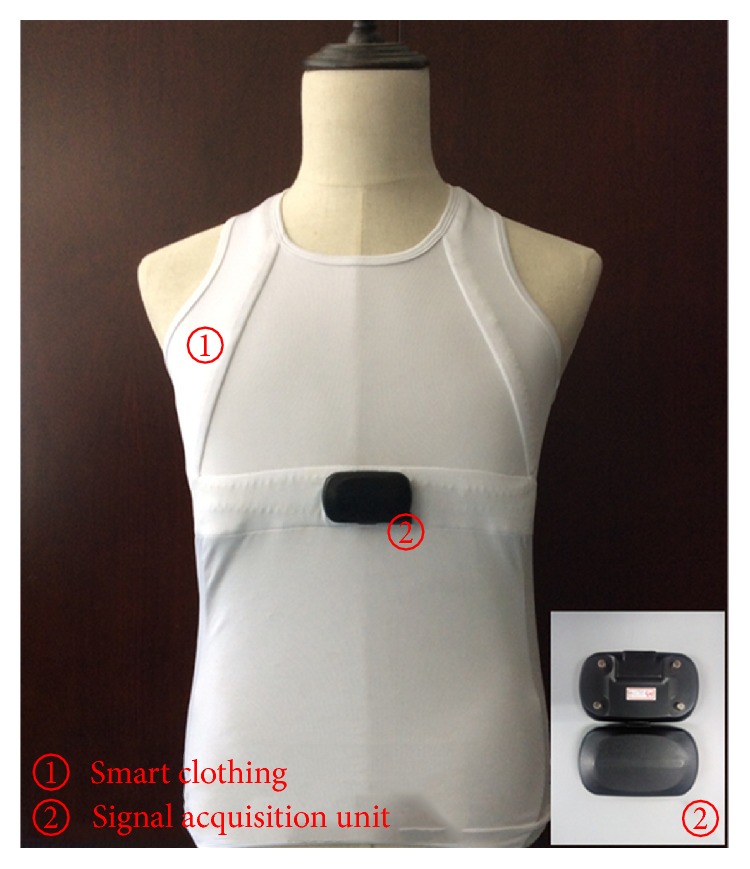
Prototype of smart clothing.

STM32F401 is used as the microprocessor unit (MPU) of the signal acquisition unit. The ECG data acquisition module, the acceleration signal acquisition module, and the BLE module are designed.  Figure 2 shows the hardware design of the smart clothing. The prototype of smart clothing is shown in Figure 3.

The signal acquisition unit is powered by a rechargeable lithium battery, which has an On-The-Go (OTG) port for power charging. When the unit is fully charged, it would wait for connection with a home gateway. Once receiving a connection request, it would respond and connect with the gateway. Then the signal acquisition unit would send data to the gateway continuously. The sampling frequency of ECG signals is 250 Hz, and the sampling frequency of acceleration signals is 100 Hz. The data package sent to the gateway is shown in Table 1. The first two bytes of the package are the header, which is used to recognize each package; the following bytes are the ECG data and the motion data.

#### 2.1.2. Home Gateway

**Figure 4:**
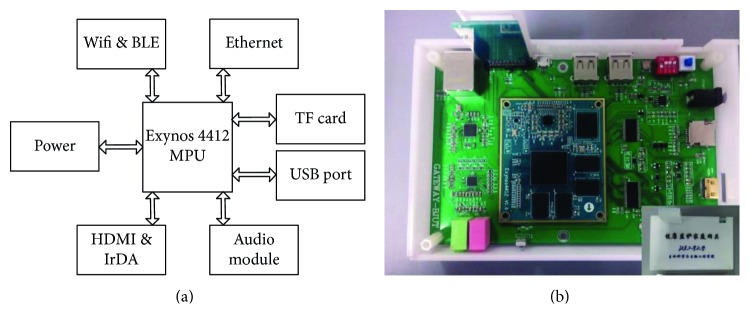
Home gateway. (a) Hardware design of home gateway. (b) Prototype of home gateway.

*(1) Hardware Design*. Exynos 4412 produced by Samsung was used as the MPU of the home gateway. It supports Linux and Android operation systems and is widely used in the consumer electronics field. To shorten the development cycle and ensure the stability of the home gateway, a kernel board is selected as the control module. An Exynos 4412, a power management integrated circuit (PMIC)—S5M8767, 1 GB flash memory, 4 GB embedded MultiMediaCard (EMMC), and an USB3503A hub controller are integrated on it. In order to make a prototype of the home gateway, the network communication modules, the power module, and other modules are also designed on the circuit board. The prototype of the home gateway is shown in Figure 4().

A 5 V power adapter is used as the main power supply of the home gateway. The kernel board functions at 4 V, so a level conversion chip is used to convert the 5 V voltage to 4 V. The S5M8767 chip on the kernel board provides different ranges of power for other modules.

The network communication modules of the home gateway include BLE, Wi-Fi for local connection, and Ethernet for remote connection. A MT6620 combo module (Wi-Fi and BLE) is selected as the local connection module, and a DM9621 module as the remote connection module.

To make it convenient for the elderly, a High-Definition Multimedia Interface (HDMI) port for TV connection and an Infrared Data Association (IrDA) port for an infrared remote control are designed. The audio module is also designed by using a WM8960 chip. It is connected to the kernel board via inter-integrated circuit sound (I2S) and inter-integrated circuit (I2C) ports. To simplify the design of the home gateway, three Universal Serial Bus (USB) ports are developed to connect a camera or other modules.

**Figure 5:**
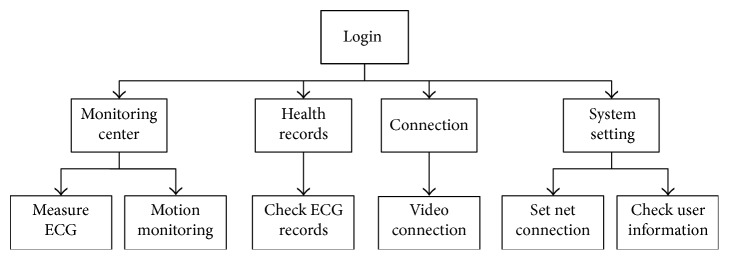
Structure of the App of home gateway.

*(2) Software Design*. Firstly, the Android operating system is transplanted onto the kernel board. Then an application (App) is developed based on the Android platform. The structure of the App is shown in Figure 5.

A key capability of home gateways is the identification of different users. Generally, typing the username and password is used to identify different users, but it is not convenient for the elderly. In the proposed system, the Bluetooth address of the smart clothing is used as the identification code (ID), which is bound to an elder. That means, by identifying different smart clothings (different Bluetooth addresses), the system can recognize different users. In order to make it easy to operate, we allocate a quick response (QR) code to each smart clothing, and the information included in the QR code is the Bluetooth address of the clothing.

The App starts and enters the login page when the gateway is powered on. The elderly can use a camera connected to the home gateway to scan the QR code of his or her smart clothing to log in. Then the login page will jump to the main menu thereupon. In the main menu, there are four modules. These are “Monitoring Center,” “Health Records,” “Connection,” and “System Settings.” The “Monitoring Center” module is used for connecting the smart clothing, adopting data, processing data, and displaying heart rate or other information. The “Health Records” module can provide the user's ECG records, motion information, and the auto-diagnosis information from the gateway. The “Connection” module is used for establishing the connection between the elderly and doctors through a camera. Doctors would contact the elderly in a regular time to confirm their health condition, and for the urgent situation, both the doctors and elderly can contact each other at any time. In the “System Setting” module, the elderly can set the net connection mode and check his basic information.

There are two main methods of remote data transmission, that is, transmitting data packets in real time and transmitting data files at regular time intervals. The first method can guarantee the server getting the real-time data, but the data packet lose is severe. The second method can reduce the packet loss rate although it cannot ensure real-time data transmission. The health care system designed in this paper adopts the second way, and a local database is constructed by using SQLite to manage the local data.

#### 2.1.3. Health Care Server

The health care server has sufficient operating capability of mining the ECG and motion data. The technologies of distributed system and cloud computing introduced in recent years make the big data processing and information mining possible. In the proposed system, the server is developed on the WAMP platform and is transplanted onto the Ali Cloud platform.

The realization of health care server includes database design and website design. The database is used for information storage, and the website is used to realize the functions of managing user information, reading data files, and so on. PHP language is used to develop the website.

MySQL is chosen to design a database to store the information of the elderly and doctors. Four tables entitled “User,” “Doctor,” “UserData,” and “UserDiag” are built. Table “User” and table “Doctor” are used to store the basic information of the elderly and doctors. Table “UserData” stores the index of ECG files, and table “UserDiag” stores the doctors' diagnostic information.

**Figure 6:**
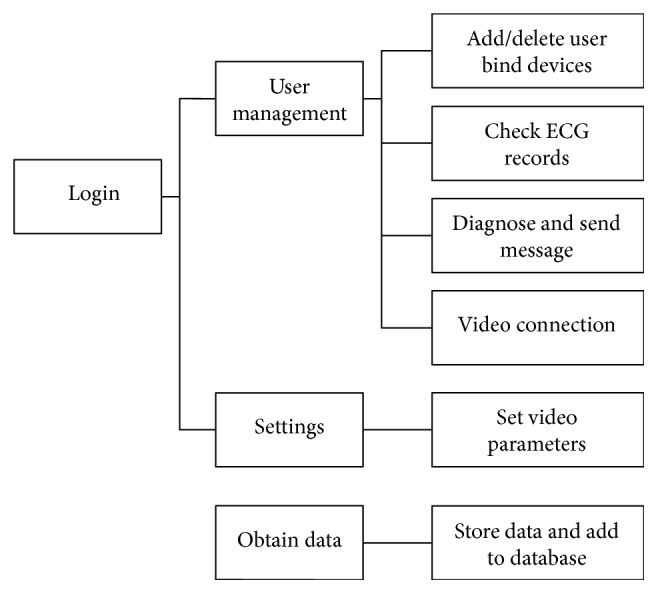
Website design.

Figure 6 shows the functions of the website, including receiving data files from the home gateway, saving data files, and managing the database according to users' information. When the doctors log in to the website, they can add/delete users or allocate a smart clothing to an elder. They can also read ECG records, make diagnoses based on the ECG records, mark the ECG graph, send diagnostic information or advice to the home gateway, and accept video connection requests from the elderly.

### 2.2. Data Compression Algorithm

The ECG sampling frequency of the signal acquisition unit in the smart clothing is 250 Hz with 12-bit resolution over a −5.27 mV to 5.27 mV range, so the data quantity of three leads is 4050000 bytes per hour. Thus, it is a high load for the home gateway to store or transmit the data. The most direct and effective way to reduce the resource consumption of the home gateway is to compress the data.

**Figure 7:**
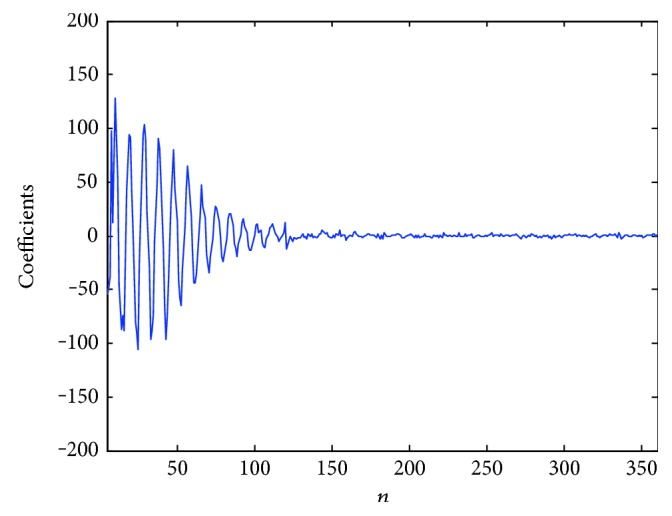
DCT of ECG (number 100).

Data redundancy would be caused if the sampling rate is too high when a signal is decimated. Reducing the sampling rate can decrease the data quantity. The main frequency spectrum of the ECG signal ranges from 0.05 Hz to 50 Hz. If the sampling frequency is greater than or equal to 200 Hz, this signal can be downsampled by factor 2, which can decrease the data quantity and keep the frequency range at the same time [12]. The original signal can be reconstructed from the downsampled signal by conducting the interpolation process with acceptable distortion. Discrete cosine transform (DCT) can transform a signal to the frequency domain and preserve the real number only. In addition, the DCT has the property of energy compaction, and the ECG signal information tends to be concentrated in a few low-frequency components of its DCT signal, which can be seen in Figure 7. According to this, the high-frequency part of DCT signal can be abandoned to decrease the number of ECG data. The method of combining the sampling rate conversion and DCT is used in the design to compress the ECG data.

**Figure 8:**
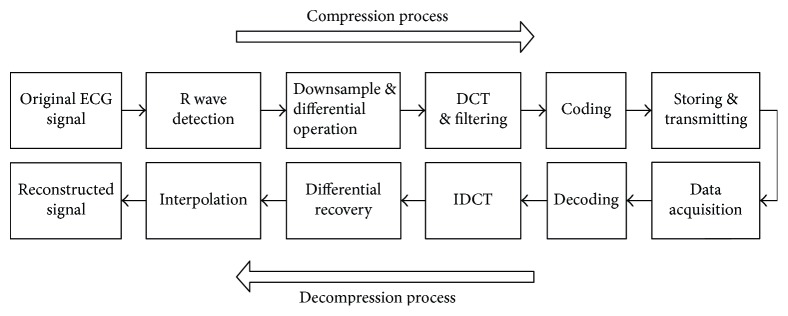
Process of data compression and decompression.

The process of the data compression is as follows (Figure 8): step 1—detect the *R* wave locations of ECG signal and choose the data from one *R* wave location to its next *R* wave location as the original signal. The *R* wave locations were detected by using the Pan-Tompkins algorithm [17]. Step 2—decimate the original signal by factor 2 and conduct forward-differential operation. Step 3—get the linear transformation (DCT) of the differential signal. It can be seen that most coefficients are nearly zero from 20% of the signal to the last. So we conserve the first 20% points of the DCT signal (20% filtering window). Step 4—code the filtered DCT signal. The data are encoded by using a fixed encoding table according to the probability of all filtered DCT data, rather than using the Huffman coding method which uses dynamic encoding tables. When decoding, the conserved DCT data are recovered according to the encoding table, and the whole DCT signal was recovered by filling zero at the end of the recovered signal in the previous step. Then the downsampled data are recovered from the reconstructed DCT data, and the original ECG signals are reconstructed by data interpolation.

Data from the MIT-BIH Arrhythmia database (acquired at 360 Hz) and several ECG records collected by using the smart clothing are employed to evaluate the ECG compression algorithm. Compression ratio (CR), distortion rate (DR), and quality score (QS) are used to evaluate the algorithm. CR is the ratio of the data amount before and after compressing, which reflects the decreasing ratio of data after implementing the algorithm. DR refers to the distortion ratio of the reconstructed signal after compression, which is usually measured by percent root-mean-square difference (PRD). QS is the ratio of CR to PRD. The higher the QS is, the higher the CR is and the lower the DR is. The three parameters are computed by

(1) $$\begin{array}{c} CR=\frac{N_{original}}{N_{reconstructed}}, \end{array}$$

(2) $$\begin{array}{c} PRD=\sqrt{\overset{n}{\underset{i=1}{\sum }}{\left(X\left(i\right)-\hat{X}\left(i\right)\right)}^{2}/\overset{n}{\underset{i=1}{\sum }}{\left(X\left(i\right)\right)}^{2}}, \end{array}$$

where *X*(*i*) denotes the raw data and $\hat{X}\left(i\right)$ is the compressed data.

(3) $$\begin{array}{c} QS=\frac{CR}{PRD}. \end{array}$$

## 3. Results and Discussion

### 3.1. System Demonstration

**Figure 9:**
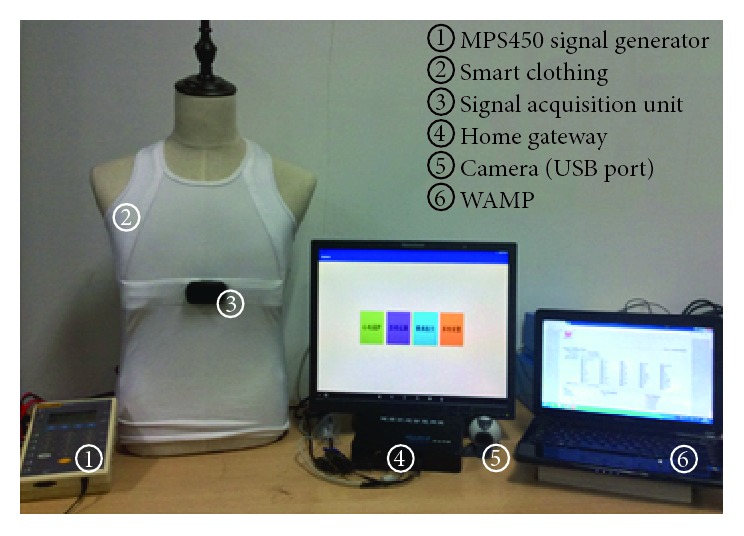
Testing environment of the system.

To validate the functionality of the whole system, a test environment is established (shown in Figure 9) and several demonstrations of the proposed system are presented. The MPS450 ECG signals generator is used to generate three-lead ECG signals. The smart clothing is connected to the MPS450 to simulate the process of signal collection. The home gateway is attached to a PC monitor through the HDMI port to simulate the digital TV. It is connected to the Internet via Wi-Fi. A web browser on another PC and a smart phone are used to test the functions of the health care server. The system demonstration includes three main parts, that is, the demonstration of local data transmission, the demonstration of remote data transmission, and the demonstration of video communication function.

**Figure 10:**
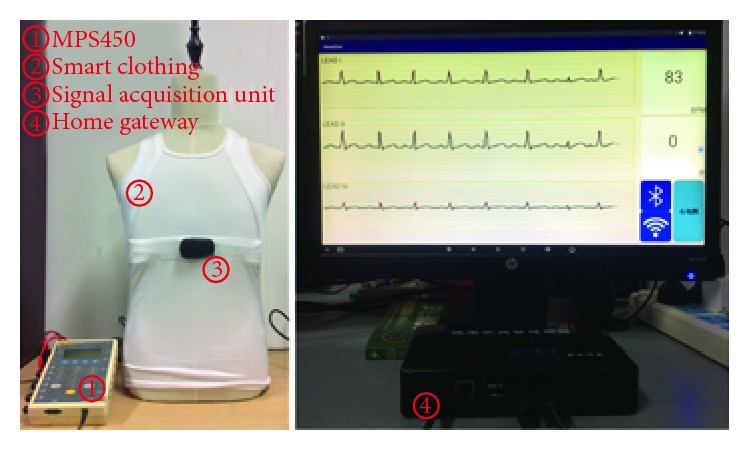
Data transmission test of smart clothing and home gateway.

**Figure 11:**
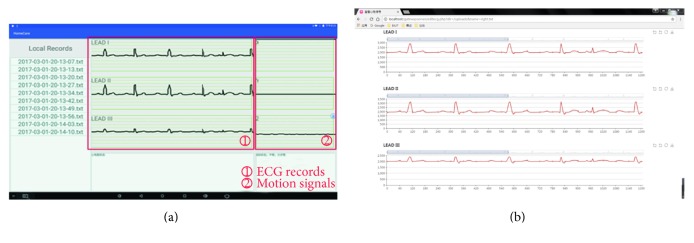
Remote data transmission test. (a) ECG and motion signals displayed by gateway. (b) ECG records displayed by a webpage.

**Figure 12:**
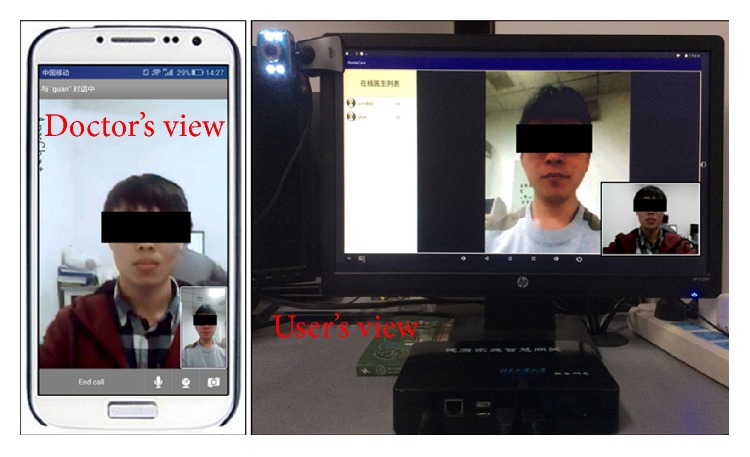
Video communication test.

Figure 10 shows the validation of local data transmission. On the left side of the figure is the MPS450 ECG signal generator; the smart clothing is connected to it. On the right side are the home gateway and the monitor. The monitor displays the ECG signal collected from the smart clothing in real-time. In the process of long-term ECG data acquisition, the delay of signals is ignorable. We have conducted an experiment of data collecting for 6 hours, and the result shows no data lost during the process. Apart from the ECG graph, the heart rate and the number of steps of the elderly are calculated and shown at the right side of the ECG graph.  Figure 11 shows the demonstration of remote data transmission. The home gateway transmits data files to the server by the Hyper Text Transfer Protocol (HTTP).  Figure 11(a) is the ECG graph and the motion signals displayed by home gateway.  Figure 11(b) is the ECG signal displayed by a web browser, the data are read from the database of the health care server. It can be found that the ECG graph in Figure 11(a) is the same as the ECG graph in Figure 11(b). The demonstration of video communication is shown in Figure 12. The smart phone on the left side shows the image from the doctors' view; the home gateway displays the corresponding image on the smart phone.

### 3.2. Data Compression Evaluation

To evaluate the proposed ECG compression algorithm, the first 36000 points (100 s) of each record in MIT-BIH Arrhythmia database are used. The CR, PRD, QS, and the time consumption of each record is shown in Table 2. Compared with the run length encoding method [11], the DCT-based [12, 15] method and the empirical mode decomposition- (EMD-) based method [14, 16] are shown in Table 3.  Figure 3() shows the comparison between the original ECG signal and the reconstructed signal of the number 100 record.

**Figure 13:**
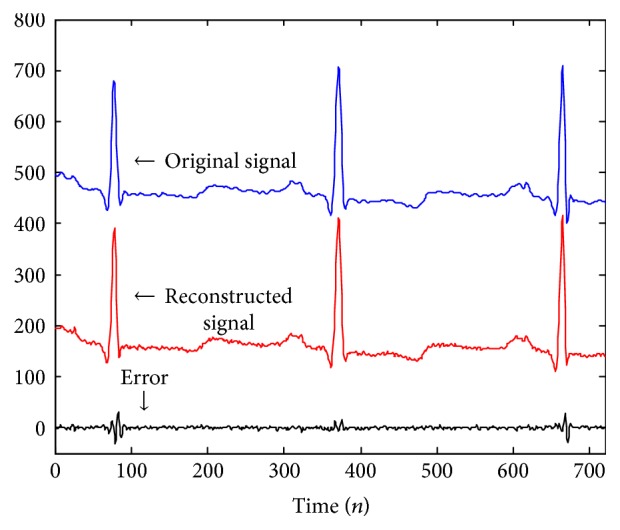
Comparison of original and reconstructed signals (number 100).

It can be seen in Table 3 that the average CR of ECG signals is 12.47 when the first 20% points of DCT signal is conserved (filtered by a 20% window) and the PRD can be controlled around 1.04%. The CR of the proposed method is higher than those of the EMD-based [16] method and the DCT-based [12] method without filtering. The CRs of EMD-wavelet-based [14] method and DCT-based (with 20% filtering window) method [15] are higher than that of the proposed method in this paper, but the PRDs of such two methods are over 2%, which is unsuitable for diagnosing [15]. Also, the run length encoding [11] method has lower CR and higher PRD. In the test, the average processing time is 290 ms, which is suitable for the real-time storage and transmission. The original signal, the reconstructed signal, and the error of reconstruction of the number 100 ECG record are shown in Figure 13. It can be seen that the distortion ratio is low and the error is around zero, demonstrating that the proposed algorithm is effective.

Also, in order to verify the effectiveness of the algorithm in a real scenario, experiments of using data acquired by smart clothing were conducted. Each ECG record was collected by 10 seconds (2500 points), and the test results are listed in Table 4. As shown in Table 4, the average CR is 11.39, and the average PRD is controlled below 2%, which demonstrates that the proposed algorithm is effective.

## 4. Conclusions

In this paper, a remote health monitoring system for the elderly based on the smart home gateway is proposed. The system has good scalability and operates easily. It can provide long-term and continuous monitoring for the elderly. In consideration of the mass data generated in the monitoring process, an ECG compression algorithm is designed. Demonstrations of the system validate that the whole system is effective and has the potential to be used in a real scenario. The test of the compression algorithm shows the possibility of applying the compression method to the real-time monitoring system.

## Figures and Tables

**Table 1:** Data packet format of the signal acquisition unit of smart clothing.

Header	Count	ECG lead I	ECG lead II	ECG lead III	*X*	*Y*	*Z*
0xA5 0xA5	Data	Data	Data	Data	Data	Data	Data

**Table 2:** Result of compression algorithm on MIT-BIH database (20% DCT window size).

Number	Evaluation parameters			
	CR	PRD (%)	QS	Time (ms)
100	12.47	1.04	11.99	481
101	12.57	0.99	12.70	300
102	12.48	0.97	12.87	285
103	12.58	1.45	8.68	292
105	12.23	0.67	18.25	294
106	12.62	1.74	7.25	291
107	11.93	1.57	7.60	316
108	12.88	0.60	21.47	241
109	12.14	0.64	18.97	299
111	12.60	0.83	15.18	269
112	12.16	0.77	15.79	275
113	12.91	1.50	8.61	301
114	13.01	0.91	14.30	216
115	12.77	1.97	6.48	288
116	10.42	2.98	3.50	306
117	13.18	0.92	14.33	264
119	12.64	1.70	7.44	315
121	12.79	0.63	20.30	256
122	12.13	1.82	6.66	289
123	13.11	1.52	8.63	298
124	13.21	1.52	8.69	302
200	12.01	1.20	10.01	294
201	11.98	0.61	19.64	272
202	13.10	0.55	23.82	252
203	11.56	1.42	8.14	307
207	12.72	0.62	20.52	267
208	12.08	1.10	10.98	313
209	11.95	1.44	8.30	280
210	12.06	0.70	17.23	271
212	12.02	1.28	9.39	297
214	12.33	1.01	12.21	289
215	11.44	1.28	8.94	302
217	12.47	1.11	11.23	314
219	12.33	1.87	6.59	294
220	12.46	2.25	5.54	290
221	12.34	0.99	12.46	287
222	12.52	0.83	15.08	251
223	12.3	1.26	9.76	292
228	12.38	0.72	17.19	284
230	12.34	1.91	6.46	293
231	12.81	1.37	9.35	307
232	12.64	0.80	15.80	223
233	11.39	1.45	7.86	309
234	12.00	1.00	12.00	282

**Table 3:** Comparison of different compression algorithm (number 100).

Compression algorithm	CR	PRD (%)	QS
EMD [16]	8.86	15.69	0.56
EMD + wavelet [14]	18.16	7.25	2.50
DCT (20% filtering window) [15]	17.21	1.95	8.83
DCT + Huffman [12]	5.14	0.65	7.91
Adaptive run length encoding [11]	5.86	4.08	1.44
Proposed (20% filtering window)	12.47	1.04	11.99

**Table 4:** Result of compression algorithm on data collected by the smart clothing.

Record	Evaluation parameters		
	CR	PRD (%)	QS
001	11.58	1.65	7.03
002	11.19	1.72	6.50
003	10.85	2.19	4.97
004	11.69	1.58	7.39
005	11.72	1.55	7.55
006	11.29	1.65	6.86
007	11.26	2.17	5.20
008	11.59	1.59	7.31
009	11.35	1.76	6.45
Average	11.39	1.76	6.58
